# Psychological aspects of body perception in depression with non-suicidal self-injury

**DOI:** 10.1192/j.eurpsy.2022.692

**Published:** 2022-09-01

**Authors:** S. Enikolopov, T. Medvedeva, O. Vorontsova, O. Boyko, D. Zhabina

**Affiliations:** Federal State Budgetary Scientific institution “Mental health research center”, Clinical Psychology, Moscow, Russian Federation

**Keywords:** body perception, depersonalization, Depression, self-harm

## Abstract

**Introduction:**

Emotional regulation appears to be a key factor in self-injury. But body image also may play an important role in self-harming.

**Objectives:**

Analysis of the relationship between non-suicidal self-injurious behavior and various aspects of body representation and body perception in adolescents and young women suffering from depression.

**Methods:**

The study involved 85 women with endogenous depression. The answer to the question “Sometimes I purposely injure myself” was used as an indicator of self-harm. The methods include: SCL-90-R, Body Investment Scale (BIS), Physical Appearance Comparison Scale-Revised (PACS-R), Body Satisfaction Scale (BSS), Cambridge Depersonalization Scale (CDS).

**Results:**

The relationship between self-injurious behavior and emotional, cognitive and behavioral characteristics of the self-body perception was revealed: more negative body image - dissatisfaction with its parts and the whole body (correlation with BSS_head ,238*, BSS_body ,472**, BSS_total_score ,453**), which is accompanied by behavioral manifestations - reduced “Protection” (correlation with BIS -,281**), higher rates of self-surveillance and comparisons of the self-body with others (PACS-R ,323**), depersonalization (CDS ,301**), body dissociation (CDS ABE ,346**), somatization (SCL-90-R ,226*).

**Conclusions:**

For young women with depression, it has been shown that when self-harming, the self-body is “devalued”, perceived as “bad,” and the need to protect it is ignored. The severity of self-harm directly correlates with the phenomena of somatopsychic depersonalization. The results obtained may indicate that rejection of the self-body, “alienated” attitude and deprivation of the body of “subjectivity” can contribute to its use as a tool for solving psychological problems, which is a risk factor for the development, consolidation and aggravation of self-injurious behavior.

**Disclosure:**

No significant relationships.

